# SJP-L-5, a novel small-molecule compound, inhibits HIV-1 infection by blocking viral DNA nuclear entry

**DOI:** 10.1186/s12866-015-0605-3

**Published:** 2015-12-02

**Authors:** Ru Bai, Xing-Jie Zhang, Yan-Li Li, Jing-Ping Liu, Hong-Bin Zhang, Wei-Lie Xiao, Jian-Xin Pu, Han-Dong Sun, Yong-Tang Zheng, Li-Xin Liu

**Affiliations:** College of Life Sciences, University of Chinese Academy of Sciences, Beijing, 100049 P. R. China; Sun Yat-Sen University, Guangzhou, 510275 P. R. China; Key Laboratory of Animal Models and Human Disease Mechanisms of the Chinese Academy of Sciences and the Kunming Institute of Zoology of the Chinese Academy of Sciences, Kunming, 650223 P. R. China; State Key Laboratory of Phytochemistry and Plant Resources in West China, Kunming Institute of Botany, Chinese Academy of Sciences, Kunming, 650204 P. R. China; Key Laboratory of Medicinal Chemistry for Natural Resources, Ministry of Education, School of Chemical Science and Technology, Yunnan University, Kunming, 650091 P. R. China

**Keywords:** HIV-1, Pre-integration complex, Nuclear entry, Capsid, SJP-L-5

## Abstract

**Background:**

Small-molecule compounds that inhibit human immunodeficiency virus type 1 (HIV-1) infection can be used not only as drug candidates, but also as reagents to dissect the life cycle of the virus. Thus, it is desirable to have an arsenal of such compounds that inhibit HIV-1 infection by various mechanisms. Until now, only a few small-molecule compounds that inhibit nuclear entry of viral DNA have been documented.

**Results:**

We identified a novel, small-molecule compound, SJP-L-5, that inhibits HIV-1 infection. SJP-L-5 is a nitrogen-containing, biphenyl compound whose synthesis was based on the dibenzocyclooctadiene lignan gomisin M2, an anti-HIV bioactive compound isolated from *Schisandra micrantha* A. C. Smith. SJP-L-5 displayed relatively low cytotoxicity (50 % cytoxicity concentrations were greater than 200 μg/ml) and high antiviral activity against a variety of HIV strains (50 % effective concentrations (EC_50_)) of HIV-1 laboratory-adapted strains ranged from 0.16–0.97 μg/ml; EC_50_s of primary isolates ranged from 1.96–5.33 μg/ml). Analyses of the viral DNA synthesis indicated that SJP-L-5 specifically blocks the entry of the HIV-1 pre-integration complex (PIC) into the nucleus. Further results implicated that SJP-L-5 inhibits the disassembly of HIV-1 particulate capsid in the cytoplasm of the infected cells.

**Conclusions:**

SJP-L-5 is a novel small-molecule compound that inhibits HIV-1 nuclear entry by blocking the disassembly of the viral core.

**Electronic supplementary material:**

The online version of this article (doi:10.1186/s12866-015-0605-3) contains supplementary material, which is available to authorized users.

## Background

The development of highly active antiretroviral therapy (HAART) in 1996 was a huge breakthrough in the treatment of human immunodeficiency virus type 1 (HIV-1) infections [1]. The combination of a three-drug therapy (cocktails) that target the viral reverse transcriptase and protease effectively reduce the viral loads of patients and prolonged their lives [2]. However, HAART has limitations. It cannot eradicate HIV-1, and there are problems with drug toxicity, as well as the emergence of drug-resistant strains [3]. These issues highlight the need to discover and develop new classes of HIV-1 inhibitors.

The life cycle of HIV-1 contains a number of distinct steps that can be used as targets for therapy. HIV-1 undergoes various steps in the host cell, including receptor binding, fusion, uncoating, reverse transcription, nuclear import, integration, transcription, translation, assembly, release and maturation to generate infective progeny. Theoretically, agents that can interfere with any of the steps of viral replication could be a valued addition to the therapeutic arsenal.

Currently, a variety of compounds have been found that can inhibit HIV-1 replication, including viral fusion inhibitors, reverse transcriptase inhibitors, protease inhibitors, and integrase inhibitors [4–8]. During the early stages of infection, HIV-1 has to transport its PIC into the nucleus for integration of the viral genome into the host DNA. This critical step for HIV-1 has attracted increasing interest as a potential drug target. It is believed that the PIC enters the nucleoplasm by passing through nuclear pore complexes (NPCs), which are composed of over 30 different nucleoporins that form stable channels in the nuclear envelope [9]. Many of the viral elements found in association with the PIC have been proposed to be important for HIV-1 nuclear import. The nuclear localization signals (NLSs) present in the viral matrix (MA) and integrase (IN) proteins, as well as various non-canonical karyophilic signals in the viral protein regulatory (Vpr), have been proposed to recruit cellular nuclear transport proteins that facilitate entry of the PIC into the nucleus [9–12]. The triple-stranded DNA flap, a cDNA intermediate of reverse transcription, was suggested to indirectly influence viral nuclear import [13]. Recently, a handful of reports suggested that the viral capsid (CA) protein plays important roles in nuclear import [14, 15]. Although the nuclear import step presents an important target for anti-viral therapeutic intervention, there is still no approved drug that targets the nuclear import mechanism.

In this study, we identified a new nitrogen-containing biphenyl compound, SJP-L-5, which can effectively inhibit both laboratory-adapted HIV-1 strains and primary isolates infection in C8166 cells and peripheral blood mononuclear cells (PBMCs). The synthesis of this new compound was based on an anti-HIV-1 bioactive dibenzocyclooctadiene lignan, gomisin M2 (SM-10), from *Schisandra micrantha* A. C. Smith. SJP-L-5 displays relatively low cytotoxicity [16]. Further analyses showed that SJP-L-5 effectively and specifically inhibits HIV-1 replication in the pre-integration stage of the viral life cycle. It blocks viral PIC nuclear import by inhibiting viral capsid uncoating, without inhibiting the function of NLS in viral proteins. Unlike other reported capsid disassembly inhibitors, SJP-L-5 does not inhibit viral reverse transcription. Thus, the unique feature of SJP-L-5 makes this new compound not only a promising therapeutic candidate in the future, but also provides a novel tool to understand the post-entry, pre-integration events in HIV-1 infection.

## Results

### Cytotoxicity and antiviral activity of SJP-L-5

**Fig. 1:**
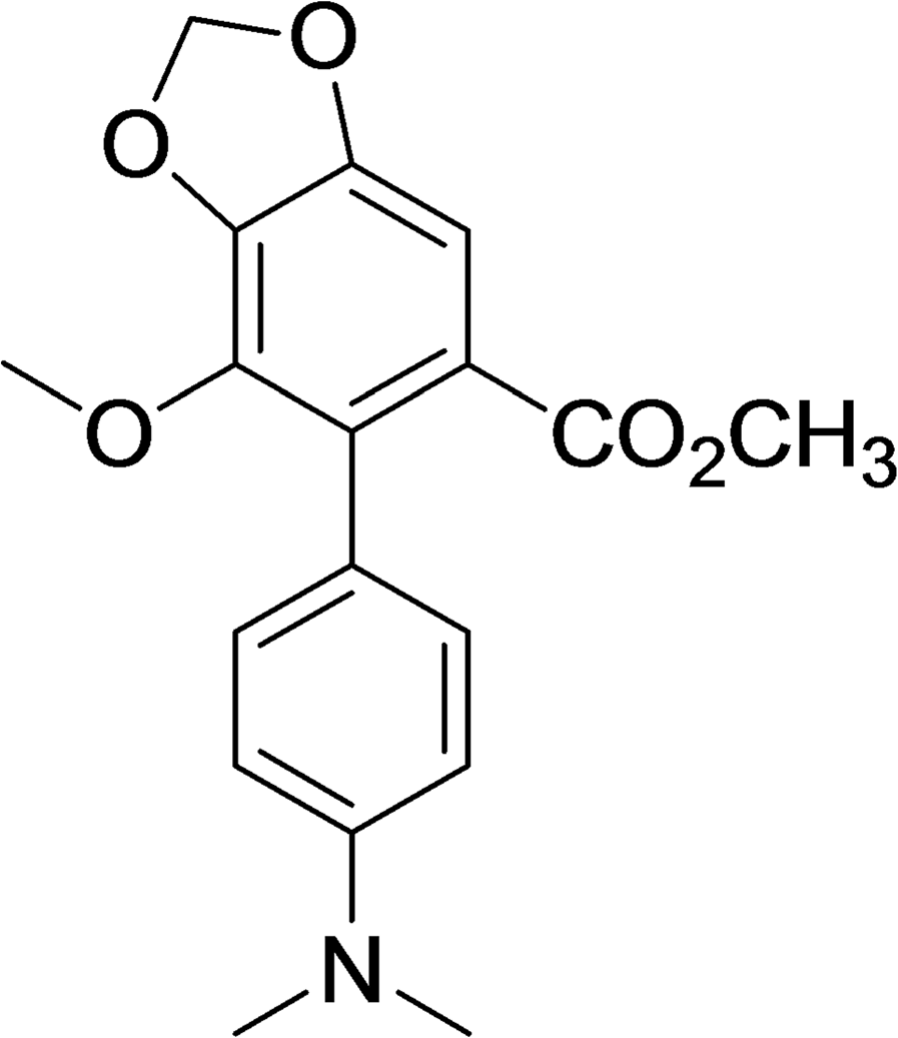
Structure of SJP-L-5. SJP-L-5 is a nitrogen-containing biphenyl compound synthesized on the basis of the dibenzocyclooctadiene lignan, gomisin M2, an anti-HIV-1 bioactive compound isolated from *S. micrantha* A. C. Smith

**Fig. 2:**
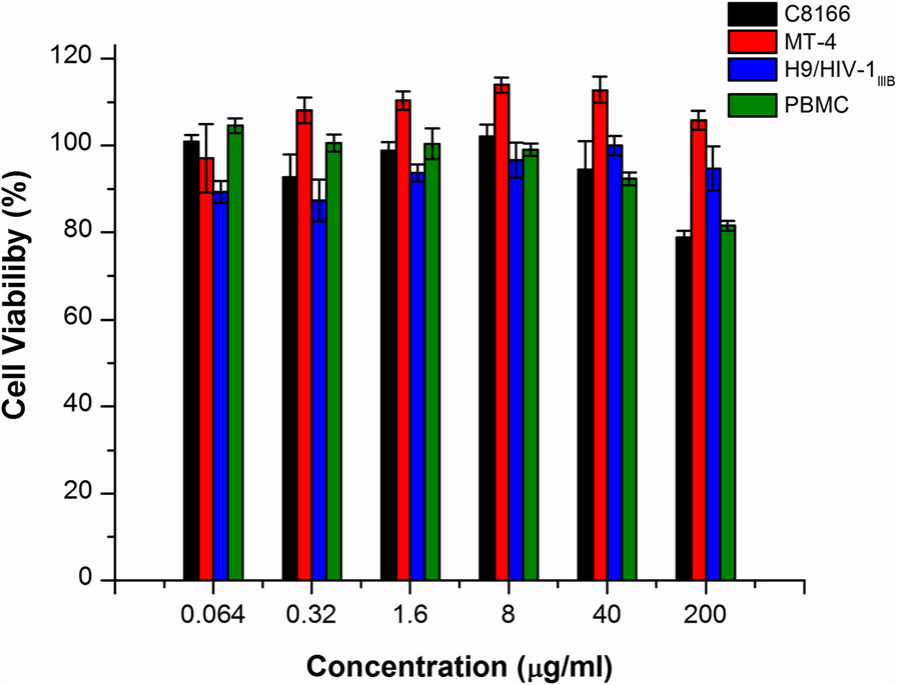
Cytotoxicity of SJP-L-5 in C8166, MT-4, H9/HIV-1_IIIB_, and PBMC cells using the MTT colorimetric method. SJP-L-5 showed low cytotoxicity to all four cell types. The CC_50_ of these cells was >200 μg/ml, respectively

**Fig. 3:**
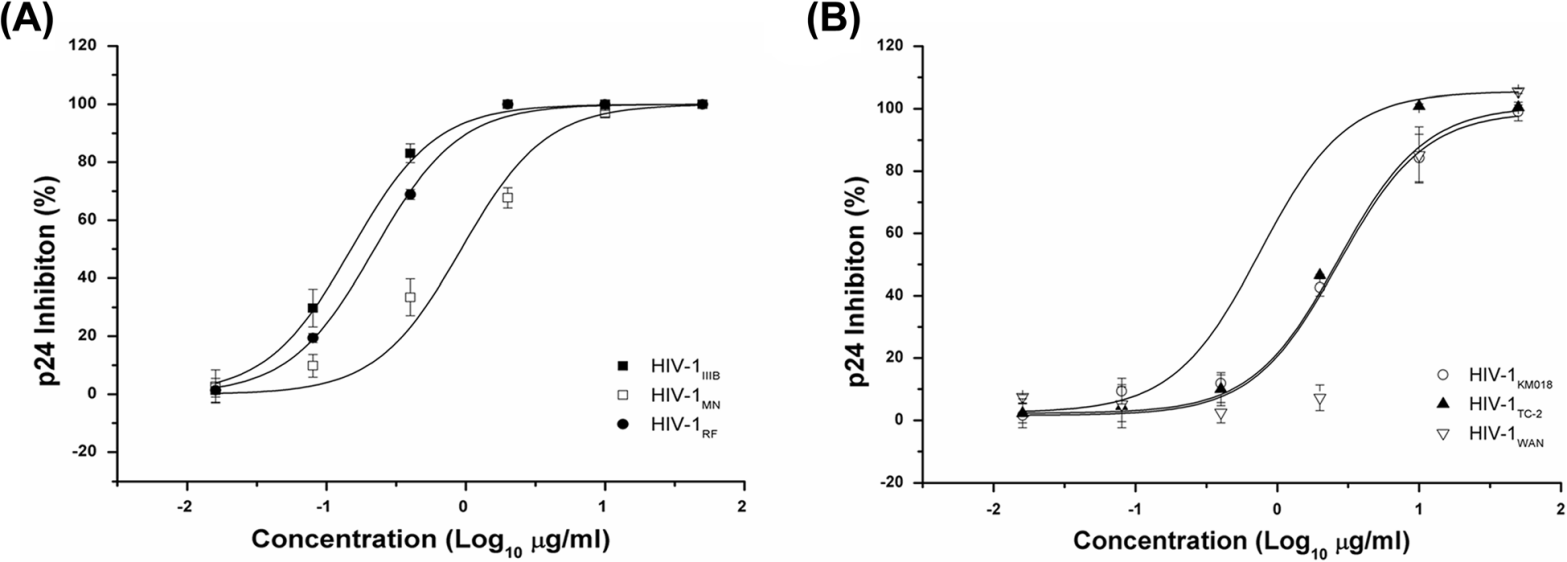
Anti-HIV-1 activity of SJP-L-5. **a** Lab-adapted strains HIV-1_IIIB_, HIV-1_MN_, and HIV-1_RF_ infect C8166 cells, and they were used to measure the antiviral activity of SJP-L-5. The EC_50_s of these strains ranged from 0.16–0.97 μg/ml. **b** Clinical isolates HIV-1_KM018_, HIV-1_TC-2_, and HIV-1_WAN_ are used to infect PBMC cells and were measured the antiviral activity of SJP-L-5. The EC_50_s of these primary isolates ranged from 1.96–5.33 μg/ml

SJP-L-5 was synthesized based on the anti-HIV-1 bioactive dibenzocyclooctadiene lignan, gomisin M2 (SM-10), from *S. micrantha* A. C. Smith (Fig. 1). To evaluate the toxicity of SJP-L-5 toward different cell lines and primary cells, 3-(4, 5-Dimethyl-2-thiazolyl)-2, 5-diphenyl-2H-tetrazolium bromide (MTT) methods were used. The 50 % cytotoxicity concentrations (CC_50_s) for SJP-L-5 in C8166, MT-4, H9/HIV-1_IIIB_, and PBMC cells were higher than 200 μg/ml (Fig. 2). To investigate the antiviral activity of SJP-L-5, HIV-1 laboratory-adapted strains (HIV-1IIIB, HIV-1MN, and HIV-1RF) and primary isolates (HIV-1 KM018, HIV-1WAN, and HIV-1TC-2) were used to infect C8166 (Fig. 3a) and PBMC cells (Fig. 3b), respectively. SJP-L-5 was a potent inhibitor of HIV-1_IIIB_, HIV-1_MN_, and HIV-1_RF_ (Table 1), and its EC_50_ values were ranged from 0.16 to 0.97 μg/ml. It also inhibited the primary isolates HIV-1_KM018_, HIV-1_WAN_, and HIV-1_TC-2_, which are prevalent in China, with EC_50_s ranging from 1.96 to 5.33 μg/ml (Table 1). After the viral inhibition efficiency and cytotoxicity of SJP-L-5 were identified, a pseudotyped virus system was used to investigate the molecule’s anti-viral mechanism.

**Table 1:** Anti-HIV-1 activity of SJP-L-5

Strains^*a*^	Subtypes	Tropisms	EC_50_ (Mean ± SD)^*b*^	
			SJP-L-5(μg/ml)	AZT^*c*^(ng/ml)
HIV-1_IIIB_	B	X4	0.16 ± 0.03	29.7 ± 9.7
HIV-1_MN_	B	X4	0.97 ± 0.13	0.9 ± 0.1
HIV-1_RF_	B	X4	0.18 ± 0.04	80.0 ± 25.5
HIV-1_KM018_	CRF07_BC	R5	2.32 ± 0.41	37.7 ± 15.1
HIV-1_TC-2_	CRF01_AE	X4	1.96 ± 0.22	5.0 ± 1.6
HIV-1_WAN_	CRF07_BC/CRF01_AE	X4	5.33 ± 0.85	4.4 ± 1.5

^*a*^HIV-1_IIIB_, HIV-1_MN_ and HIV-1_RF_ are lab-adapted strains that infect C8166 cells, and they were used to measure the antiviral activity of SJP-L-5; HIV-1_KM018_, HIV-1_TC-2_, and HIV-1_WAN_ are clinical isolates that infect PBMC cells, and they were also used to measure the antiviral activity of SJP-L-5

^*b*^n ≥ 3

^*c*^AZT, azidothymidine

### SJP-L-5 specifically inhibits HIV-1 infection at the pre-integration stage

**Fig. 4:**
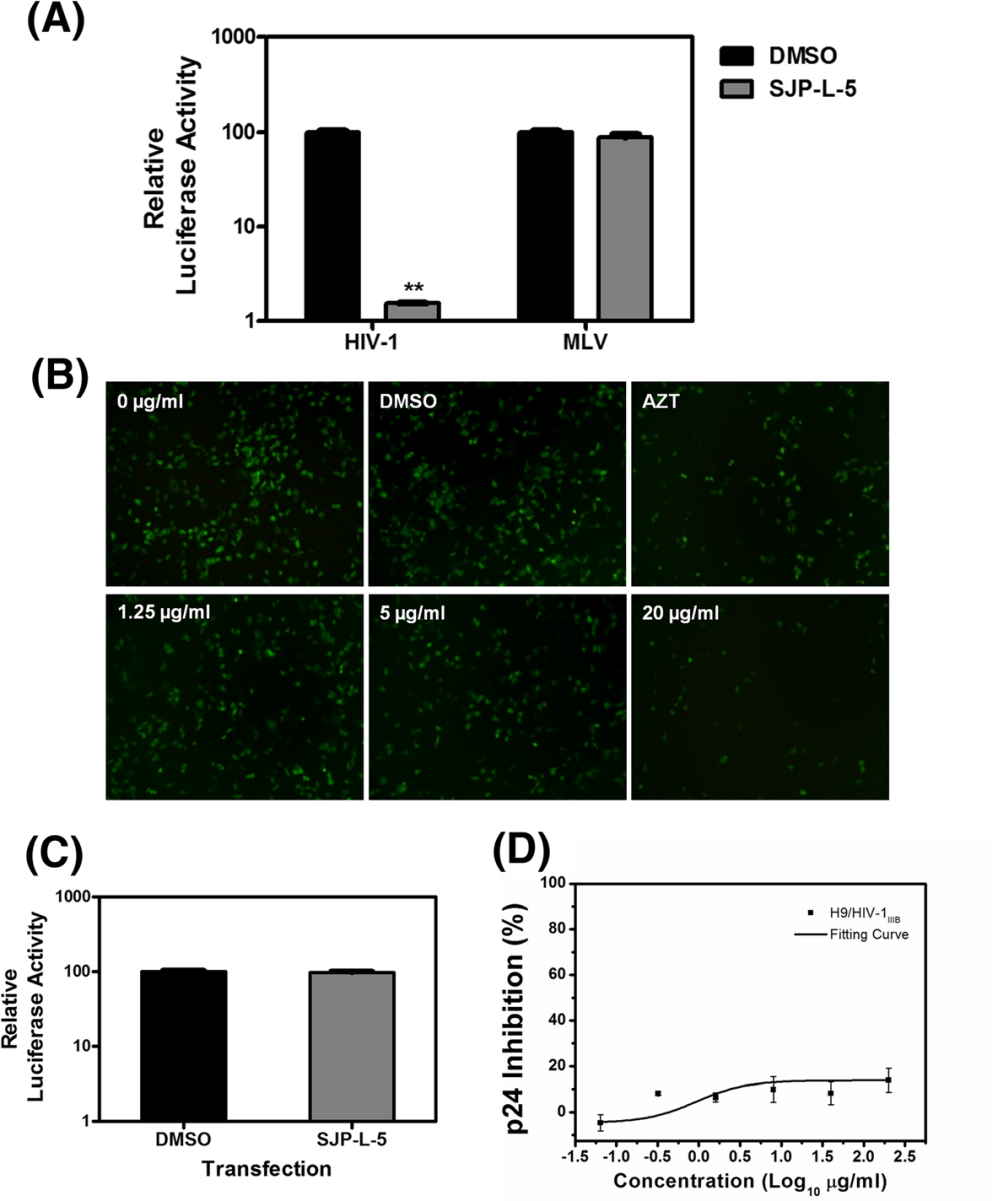
SJP-L-5 specifically inhibits HIV-1 at the pre-integration stage. **a** 293 T cells were infected with VSV-G pseudotyped HIV-1-Luc or MLV-Luc in the presence of SJP-L-5 (5 μg/ml, Gray) or DMSO (Black) as a negative control. The antiretroviral activity was evaluated by testing the activity of the luciferase reporter gene at 48 h post infection. Error bars indicate standard deviations of triplicate values. Statistical significance was analyzed by the Student’s *t* test. ^**^ *P* < 0.01 versus the negative control. **b** 293 T cells were infected with VSV-G pseudotyped HIV-1-GFP, in the presence of SJP-L-5 (0, 1.25, 5, or 20 μg/ml), or DMSO as a negative control; AZT (10 μM) was used as a positive control. 24 h after infection, infectivity was analyzed with a fluorescence microscope. **c** 293 T cells were transfected with 0.1 μg of pNL4-3luc in the presence of SJP-L-5 (5 μg/ml, (Gray)) or DMSO (Black) as a negative control. 48 h later, cellular luciferase activity was determined. **d** The antiviral activity of SJP-L-5 was tested using H9/HIV-1_IIIB_ cells, and EC50s were greater than 200 μg/ml. The inhibition was calculated as the p24 levels in control cells divided by that in SJP-L-5 treated cells

To examine whether the inhibition of infection extends to retroviruses besides HIV-1, we assayed infection by HIV-1 and murine leukemia virus (MLV) in the presence of SJP-L-5 (20 μg/ml). 293 T cells were infected with vesicular stomatitis virus (VSV)-G pseudotyped HIV-1-luciferase (Luc) or MLV-Luc viruses. At 48 h post infection, luciferase activity was measured with a luminometer. As was shown in Fig. 4a, SJP-L-5 specifically inhibited HIV-1 infection. The viral encoded luciferase activity in SJP-L-5 treated cells was reduced to 1.5 % comparing to that in DMSO treatment cells (*P* = 0.0011, *n* = 3), while SJP-L-5 showed no effect on MLV infection. This result demonstrated the selectivity of SJP-L-5’s antiviral activity.

Since SJP-L-5 could selectively inhibit VSV-G pseudotyped HIV-1-luciferase virus infection, further experiments were conducted to identify which step of the HIV-1 life cycle was blocked by SJP-L-5. By convention, the complicated HIV-1 life cycle was divided into pre-integration and post-integration stages. The steps from receptor binding to PIC nuclear import were referred to as the pre-integration stage, and the rest of the replication steps of HIV-1 were considered to be the post-integration stage. Using assays developed by Guangxia Gao’s group [17], we infected 293 T cells with VSV-G pseudotyped HIV-1-green fluorescent protein (GFP) in the presence of different concentrations of SJP-L-5 (0, 1.25, 5, or 20 μg/ml), with DMSO as a negative control and azidothymidine (AZT) (10 μM) as a positive control. The results were observed by a fluorescence microscope (Fig. 4b), which showed that SJP-L-5 inhibited the infection of VSV-G pseudotyped HIV-1-GFP in a dose-dependent manner, consistent with the results shown in Fig. 4a. To determine whether SJP-L-5 could block the post-integration stage of the viral life cycle, a proviral luciferase reporter DNA genome bypassing the pre-integration stage of infection was transfected into 293 T cells. The results showed that the expression of the reporter from the proviral DNA was not affected by SJP-L-5 treatment compared to the DMSO treated control (*n* = 3) (Fig. 4c), suggesting that SJP-L-5 did not affect the post-integration stage of the HIV-1 life cycle, which includes viral genome integration, transcription, transport out of the nucleus, and viral protein translation. To confirm that SJP-L-5 did not affect the viral post-integration steps including HIV-1 transcription and translation, we also investigated the antiviral activity of SJP-L-5 in the chronically infected H9/HIV-1IIIB cell line which stably expresses HIV-1 _IIIB_ proviral DNA. The EC_50_s of SJP-L-5 were greater than 200 μg/ml. Furthermore, there was no significant difference in the levels of p24 (CA) between the DMSO treated control and the SJP-L-5 treated group (*n* = 3) (Fig. 4d). Taken together, these results showed that SJP-L-5 blocked HIV-1 infection at stages of pre-integration.

### SJP-L-5 blocks the nuclear entry of the pre-integration step in the HIV-1 life cycle

**Fig. 5:**
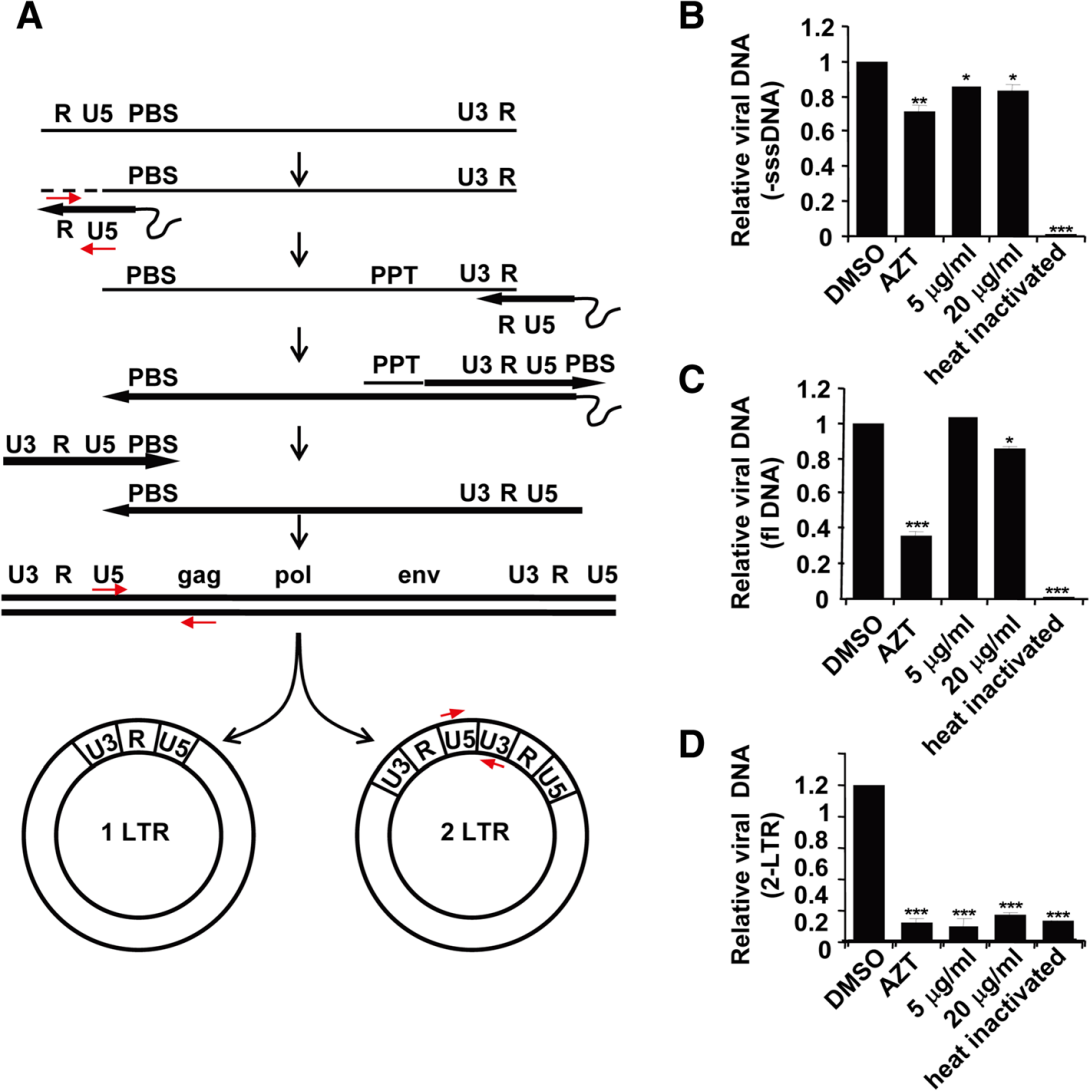
Real-time PCR analysis of the formation of HIV-1 DNAs. **a** Schematic representation of the reverse transcription and integration of HIV-1 genome. The primers used to amplify the viral DNAs were indicated. b-d. Real-time PCR analysis of HIV-1 DNA in 293 T cells. 293 T cells were infected with VSV-G pseudotyped HIV-1-GFP in the presence of DMSO (control) or SJP-L-5 at indicated concentrations. AZT (10 μM) was used as a positive control. At 24 h post infection, low-molecular-weight DNA, including the viral DNA, was isolated. The viral -sssDNA (**b**), flDNA (**c**), and 2-LTR circular DNA (**d**) levels were measured by real-time PCR. Error bars indicate standard deviations of triplicate values. Statistical significance was analyzed by the Student’s *t* test. ^*^ *P* < 0.05‚ ^**^ *P* < 0.01 and ^***^ *P* < 0.001 versus the negative control

To further explore the exact step at which SJP-L-5 blocks the pre-integration stage of the HIV-1 life cycle, 293 T cells were infected with VSV-G pseudotyped HIV-1-GFP virus, and synthesis of the viral DNA was examined by real-time PCR. Formation of a minus-strand strong-stop DNA (−sssDNA) is the first step of reverse transcription and the full-length DNA (flDNA) synthesis represents the last step of reverse transcription [18] (Fig. 5a). We detected both –sssDNA and flDNA levels in HIV-1 infected 293 T cells, with or without the treatment of SJP-L-5. SJP-L-5 treatment had little effect on the levels of -sssDNA (Fig. 5b) and flDNA (Fig. 5c), while the RT inhibitor AZT effectively reduced the flDNA levels, implicating that SJP-L-5 does not inhibit reverse transcription. To test whether SJP-L-5 inhibits virus nuclear entry, the 2-long terminal repeat circles (2-LTR) were analyzed. 2-LTR circles are the dead-end byproducts of the viral life cycle, and a conventional marker of nuclear entry [19–22] (Fig. 5a). Data showed that SJP-L-5 treatment significantly reduced the level of 2-LTR circles (Fig. 5d). The reduction in the level of 2-LTR circles suggests that SJP-L-5 interferes with the import of the PIC into the nucleus.

### SJP-L-5 does not affect the subcellular localization of HIV-1 IN, MA or Vpr protein

We next analyzed the mechanism by which SJP-L-5 blocks the nuclear entry of the PIC. Currently, the molecular mechanism that regulates the delivery of the HIV-1 PIC into the nucleus is not entirely clear. Three karyophilic viral proteins, including HIV-1IN [23–25], MA [26–31], and Vpr [32], have been reported to contain one or two NLSs that can promote the active nuclear import of the PIC. HIV-1 MA contains two subcellular localization signals: a myristoylated N-terminus governs particle assembly at the plasma membrane and an NLS that facilitates the import of the PIC into the nucleus of non-dividing cells [26–28]. These two crucial functions act at different stages of the HIV-1 life cycle. Myristoylation occurs at the N-terminus of MA and prevents the NLS-mediated transport of MA to the nucleus [26–28]. To investigate only the karyophilic function of MA in the nuclear import of the PIC, GFP was in-frame fused with the N-terminus of MA to block the myristoylation (GFP-MA). GFP was also in-frame fused with the C-terminus of MA so that the myristoylation was not affected (myrMA-GFP). We also constructed plasmids with GFP fused to the N-terminus of IN and Vpr (GFP-IN and GFP-Vpr), the primers used in the plasmids construction were listed in Additional file 1: Table S1. 293 T cells were transiently transfected with the corresponding expression plasmids. As shown in Additional file 2: Figure S1 (A), myrMA-GFP localized in the cytoplasm and associated with the plasma membrane, but was excluded from the nucleus, while GFP-MA localized in the nucleus. GFP-IN and GFP-Vpr also localized in the nucleus [see Additional file 2: Figure S1 (A)]. GFP alone localized in both the nucleus and the cytoplasm. GFP_2_-NLS, which contains an NLS sequence, localized in the nucleus. These results suggest that the GFP fusion proteins localized properly. Then, SJP-L-5 (5 μg/ml) was added to the cells to determine whether SJP-L-5 blocked the nuclear import of the PIC by interacting with these three proteins. The results showed that SJP-L-5 did not alter the subcellular distribution of the three viral proteins. A higher concentration of SJP-L-5 (20 μg/ml) also failed to alter the proteins’ localization [see Additional file 2: Figure S1 (B-D)]. These observations suggest that SJP-L-5 does not affect the transport of these three karyophilic viral proteins and that SJP-L-5 blocks the nuclear import of the PIC through mechanisms other than NLS-mediated nuclear transport.

### SJP-L-5 does not target the DNA flap

Given the important roles of DNA flap played in PIC nuclear import [13, 76], we tested whether SJP-L-5 activity targets this element. We used CMVΔR8.2-Luc (pCMVΔR8.2 encoding Gag and Gag-Pol, together with a genomic RNA encoding plasmid pHR’-Luc which lacks the flap) virus. 293 T cells were infected with VSV-G pseudotyped NL4-3-Luc which contains the flap sequence or CMVΔR8.2-Luc in the presence of SJP-L-5. At 48 h post infection, luciferase activity was measured with a luminometer. Treatment of SJP-L-5 resulted in significant inhibitions in both NL4-3luc and CMVΔR8.2-luc infection, comparing to the DMSO treated controls [see Additional file 3: Figure S2]. This result suggests that the DNA flap-mediated nuclear import was not a dominant target for SJP-L-5. The specific inhibition of HIV-1 infection, along with the results described above, led us to test whether SJP-L-5 plays a role in uncoating the viral capsid from the PIC.

### SJP-L-5 inhibits HIV-1 capsid disassembly

MLV can only infect mitosing cells, in which the nuclear envelope is dissolved during cell division. MLV keeps its CA intact when its DNA is transported to the nucleus [33]. In contrast, HIV-1 infects post-mitotic cell types, and accordingly, its viral CA shell has to be dissembled during nuclear entry. Various studies have reported the importance of HIV-1 CA in the infection of non-dividing cells [34–37].

**Fig. 6:**
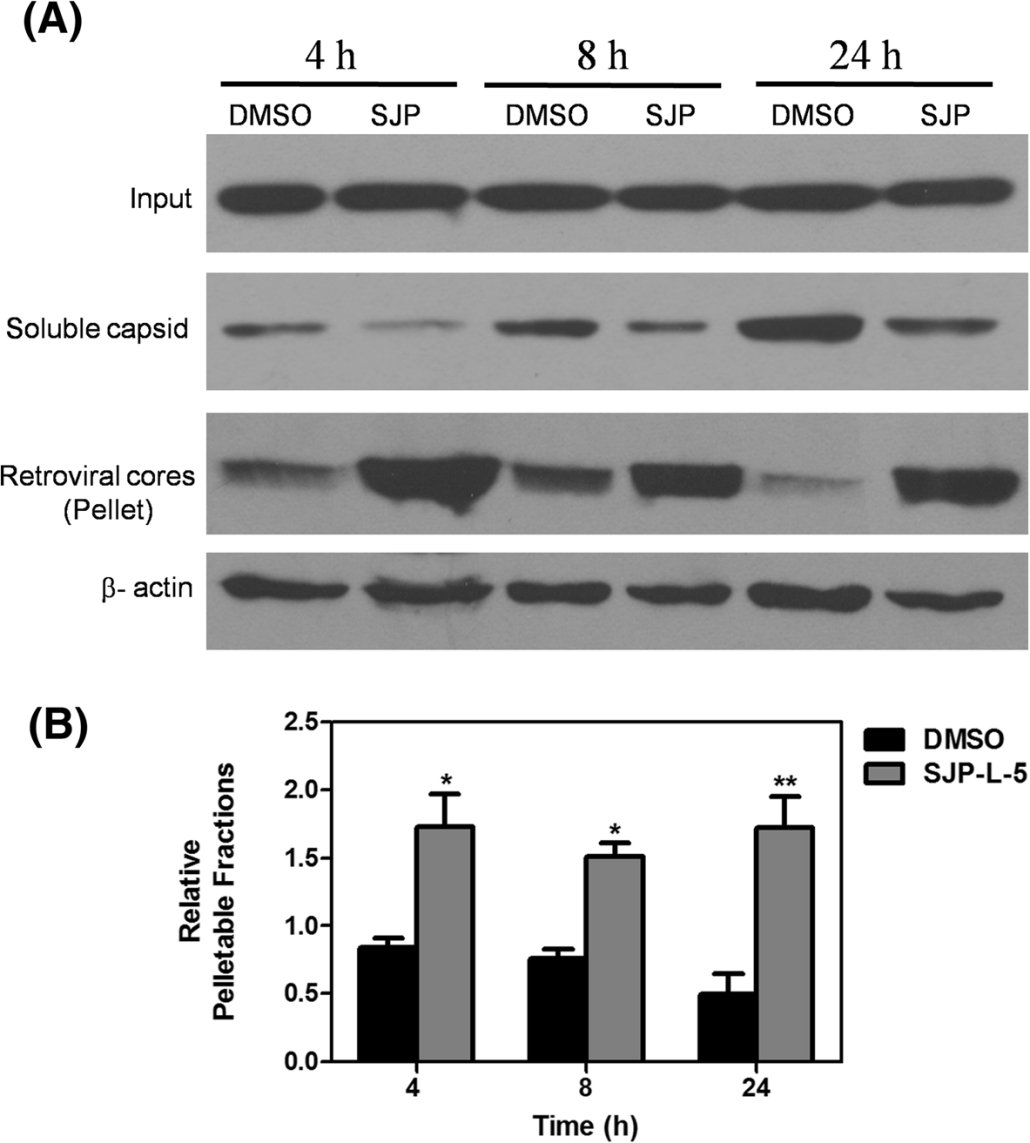
Effect of SJP-L-5 on the fate of the HIV-1 capsid in infected cells. **a** 293 T cells were infected with VSV-G pseudotyped HIV-1-GFP for 4 h in the presence of SJP-L-5 at a final concentration of 20 μg/ml, or DMSO as a negative control. At different time points post infection, the cells were collected and analyzed using the fate-of-capsid assay, as described in the Methods. The total input, soluble capsid, and pellet fractions were Western blotted using a monoclonal antibody directed against the HIV-1 p24 capsid protein. β-actin was used as pellet fraction control. **b** The quantification of the pelleted fraction relative to the input was calculated. Error bars indicate standard deviation in triplicate values. Statistical significance was analyzed by *t* test. *P < 0.05 and **P < 0.01 versus the no drug control. SJP: SJP-L-5

A fate-of-capsid assay was used to investigate whether SJP-L-5 could prevent the viral CA proteins from dissociating from PICs. This method was developed by Joseph Sodroski’s lab and was successfully used to analyze CA disassembly by evaluating the levels of particulate and soluble CA in the cytosol of infected cells [37–39]. At different time points post infection, cell lysates were layered onto a 50 % (w/v) sucrose cushion and centrifuged. The samples were collected and analyzed by Western blotting. As was shown in Fig. 6, the levels of pelleted CA in the cells treated with SJP-L-5 were higher than those in the control cells at all time points. Accordingly, the amount of soluble CA in the treated cell cytosol was lower than that in the cytosol of control cells. These data showed that SJP-L-5 inhibited viral DNA nuclear import by blocking CA disassembly.

**Fig. 7:**
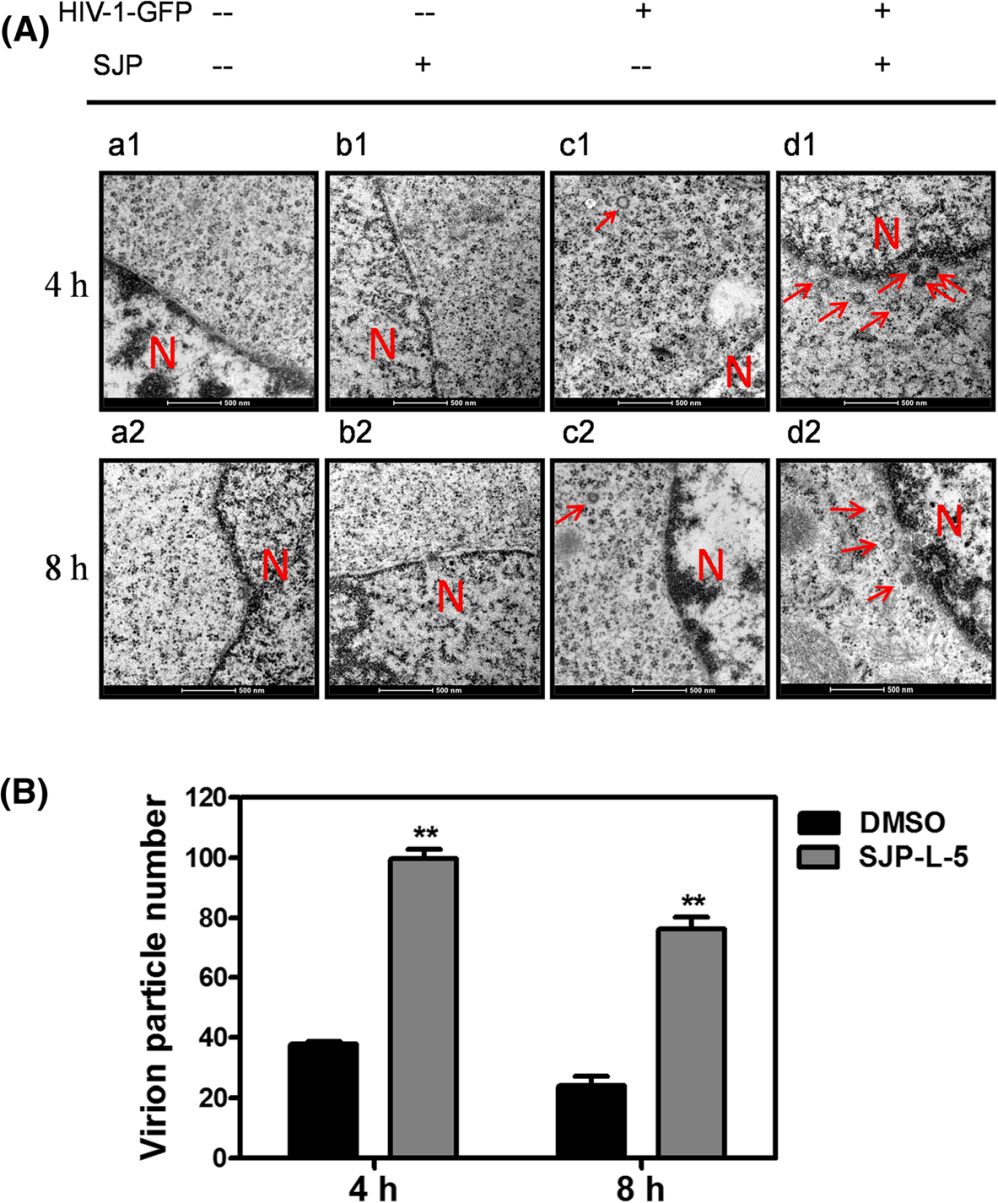
Electron micrographs of SJP-L-5-treated 293 T cells. **a** Electron micrographs of the ultrastructure of SJP-L-5-treated cells that were infected with VSV-G pseudotyped HIV-1-GFP. a_1_, a_2_: blank control. b_1_, b_2_: no HIV-1 control (293 T cells were only treated with SJP-L-5). c_1_, c_2_: DMSO was used as a negative control (293 T cells were infected by HIV-1 in the absence of SJP-L-5). d_1_, d_2_: 293 T cells were infected by HIV-1 in the presence of SJP-L-5 (20 μg/ml). N: nucleus. Arrows indicate HIV-1 particles. Scale bars: 500 nm. **b** Quantification of total HIV-1 particles in 20 fields in the DMSO and SJP-L-5 groups. Error bars indicate standard deviations of triplicate values. Statistical significance was analyzed by the Student’s *t* test. ^**^ *P* < 0.01 versus the negative control

To further verify whether SJP-L-5 could inhibit HIV-1 capsid uncoating, a transmission electron microscopy (TEM) assay was used to observe the uncoated viral core directly. Based on the results of fate-of-capsid assay, two time points (4 h and 8 h post infection) were selected for the TEM assay. In contrast to control cells, there were more capsid proteins around the nucleus in the presence of SJP-L-5 (20 μg/ml) (Fig. 7). The TEM results are consistent with those from the biochemical assay (Fig. 6). Thus, it was demonstrated that SJP-L-5 inhibits HIV-1 replication by inhibiting viral capsid dissociation from the PIC, thereby preventing nuclear entry.

## Discussion

In this study, we identified a novel, small molecule, SJP-L-5, which could effectively and efficiently inhibit the replication of both laboratory-adapted strains (HIV-1_IIIB_, HIV-1_MN_, and HIV-1_RF_) and primary isolates (HIV-1_KM018_, HIV-1_WAN_, and HIV-1_TC-2_) in C8166 and PBMC cells. Furthermore, we analyzed the stage of the viral life cycle that was blocked by SJP-L-5. The results from the step-by-step assays showed that SJP-L-5 can selectively inhibit HIV-1 replication by blocking the nuclear import of PICs (Figs. 4 and 5). Nuclear import of the HIV-1 PIC is a critical step in retrovirus replication. It is believed that various viral elements and host factors are involved in nuclear transport through the NPC. Three main viral factors affect the nuclear import of the PICs: NLS, the DNA flap, and the CA. Three viral proteins, IN, MA, and Vpr, have been reported to contain a NLS(s) and promote the nuclear import of PICs. In this study, we demonstrate that SJP-L-5 has no effect on the NLS-mediated nuclear import of the three proteins at 5 and 20 μg/ml concentrations [see Additional file 2: Figure S1 (B–D)], neither does it target the DNA flap (Additional file 3: Figure S2). Therefore, we focused on the last main factor, the CA.

Historically, the retroviruses MLV and HIV-1 were used as model systems to study retroviral nuclear import. MLV PIC contains a large amount of the CA protein [40, 41], while the CA protein is not strongly associated with the PIC of HIV-1 [42–45]. A major difference between them is their abilities to successfully infect non-dividing cells. The transport of the MLV genome into the nucleus requires the dissolution of the nuclear envelope, although the viral CA does not need to be disassembled; thus, cellular infection by MLV is highly dependent on the state of the cell cycle [33]. In contrast, HIV-1 infects post-mitotic cell types, and the viral CA, accordingly, has to be dissembled before its genome is transported into the host nucleus through an intact nuclear membrane. Various studies had reported the importance of the HIV-1 CA in infections of non-dividing cells [34–37]. Masahiro Yamashita and Michael Emerman reported that the CA was the major determinant of infection in growth-arrested cells. They reported that HIV-1 containing the MLV CA lost the ability to infect non-dividing cells, and that mutations in the HIV-1 CA could specifically reduce the infectivity of HIV-1 in non-dividing cells [36, 37]. Thus, we surmised that SJP-L-5 specifically targets HIV-1 CA disassembly pattern. Using a fate-of-capsid assay and TEM assays, we observed a reduction in soluble CA and elevated levels of particulate CAs in response to SJP-L-5 treatment (Figs. 6 and 7). Therefore, we obtained consistent results showing that SJP-L-5 blocks the import of the HIV-1 genome into the nucleus by inhibiting capsid disassembly. This result shows that the viral CA protein plays an important role in nuclear import. Yet, the full composition of the viral PIC is not known, and the composition of the PIC varies between different retroviruses. It is clear that differences in PIC composition, including the CA protein, contribute to the different behaviors of viruses in host cells.

Few other inhibitors targeting HIV-1 PIC nuclear entry have been previously described. BI-1 and BI-2, whose effects are similar to those of SJP-L-5, were reported to decrease the accumulation of 2-LTR circles, without affecting HIV-1 reverse transcription [46]. BI-2 resistant selection studies showed that substitution mutations at positions A105T and T107N in the CA protein are the most prevalent mutations, and these two mutation sites surrounded a conserved pocket (site 2), which was formed by alpha helices 3, 4, and 5 in the N-terminal domain of HIV-1 CA. BI-1 was shown to bind in this pocket by isothermal titration calorimetry (ITC), NMR, and X-ray crystallographic analyses [46]. The results of an in vitro stabilization assay showed that BI accelerated the assembly of HIV-1 CA-NC tubes and prevented the dissociation of preassembled CA-NC tubes. Very interestingly, another reported Pfizer inhibitor, PF74, harbors the same binding site in the CA_NTD_ site 2 pocket as the BI compounds have [47]. Recent research suggests that BI-2 and PF74 cannot inhibit the ability of an HIV-1 capsid-dependent nuclear import inhibitor MxB to bind in vitro assembled HIV-1 CA-NC complexes, but block the binding of an HIV-1 capsid-associating protein CPSF6 because PF74, BI-2, and CPSF6 bind to similar regions on the HIV-1 CA [48–50]. PF74 uses a similar mechanism to the one used by BI-2 to block HIV-1, but it inhibits reverse transcription [47, 51]. Together with our study, more and more evidence indicates the importance of CA in HIV-1 PIC nuclear import.

The well-known restriction factor TRIM5α family proteins [52–55] also inhibit HIV-1 replication by interacting with the viral CA protein. Two inhibition mechanisms have been identified: one is a proteasome-dependent process, while the other is proteasome-independent. In the proteasome-dependent process, TRIM5α recognizes specific retroviral CA cores and accelerates the uncoating of incoming viral capsid, this restricts HIV-1 reverse transcription, decreases the production of sssDNA, and prevents HIV-1 replication in infected cells. Some experiments [56–59] have shown that proteasome inhibitors could uncouple with TRIM5α restriction of HIV-1 reverse transcription in rhesus macaques, but the resultant HIV-1 cDNA could still not enter the nucleus. Kutluay et al. [60] showed that proteasome inhibition restored large sub-viral complexes containing CA, and Campbell et al. [61] found that proteasome inhibition leads to the accumulation of fluorescently labeled HIV-1 virions in the cytoplasm of restricted cells, which was the same phenomenon as we observed with SJP-L-5 (Figs. 6 and 7).

Also, a number of cell host proteins have been reported to be involved in the CA uncoating process and PIC nuclear entry. For example, CPSF6 could bind CA and stabilize viral cores [50, 62, 63]; Nup358/RanBP2 aids HIV-1 core to dock at NPC [64, 65]; Nup153 binds CA and participates to both HIV-1 translocation and integration steps [66]; CypA could destabilize the capsid shell during viral entry and uncoating step [67], or be a chaperon facilitating correct capsid condensation during viral maturation [68, 69]. Some studies showed that abrogation of CA binding with CypA, either through CypA depletion or competition with the small molecule cyclosporine, could rescue viruses from the Nup153 and Nup358 depletion cells [70]. The roles of these host factors will give us a clue to find the exact target site of SJP-L-5.

Further studies, including the selection of SJP-L-5-resistant variants, will be needed to explore the exact mechanism of SJP-L-5’s blockage of HIV-1 infection, as well as to shed light on the complicated HIV-1 uncoating process.

## Conclusion

In this work, we identified a new nitrogen-containing biphenyl compound, SJP-L-5 that specifically blocks HIV-1 PIC import into the nuclei of infected cells. SJP-L-5 inhibited the disassembly of the HIV-1 capsid in the cytoplasm. This novel compound could be used as a potential drug candidate for anti-HIV-1 therapy.

## Methods

### Reagents and chemicals

SJP-L-5 was provided by Professor Handong Sun (Kunming Institute of Botany, Chinese Academy of Sciences). DMSO, AZT, MTT, sodium dodecyl sulfate (SDS), N, N-dimethylformamide (DMF), phytohemagglutinin (PHA), and interleukin-2 (IL-2), were purchased from Sigma-Aldrich (St. Louis, MO, USA).

### Cells and viruses

C8166, MT-4, and H9 cells were kindly provided by the AIDS Reagent Project, the UK Medical Research Council (MRC). Laboratory-adapted strains, including HIV-1_IIIB_, HIV-1_MN_, and HIV-1_RF_ were obtained from the NIH AIDS Research and Reference Reagent Program (Bethesda, MD, USA). Clinically isolated HIV-1 strains, including HIV-1_KM018_, HIV-1_TC-2_, and HIV-1_WAN_ were isolated from local AIDS patients in Yunnan, China, prior to antiviral drug treatment (Ethical Approval Number: SWYX-2009018). PBMCs were isolated by the Ficoll-Hypaque method from whole blood collected from healthy donors (Ethical Approval Number: SWYX-2009019). Ethical approval was given by the Biomedical Ethics Committee of Kunming Institute of Zoology of the Chinese Academy of Sciences with the following reference number: SWYX-2010018 and SWYX-2010019. Informed written consent was given by the patients and donors before sampling. 293 T cells used in this study were all maintained in Dulbecco’s Modified Eagle’s Medium (DMEM) supplemented with 10 % fetal bovine serum (FBS) (Gibco, Grand Island, NY, USA) at 37 °C in a 5 % CO_2_-humidified atmosphere. C8166, MT-4, and H9/HIV-1_IIIB_ suspension cell lines were maintained in RPMI-1640 medium containing 10 % FBS, 100 U/ml of penicillin G, and 100 μg/ml of streptomycin. PBMCs were stimulated with 5 μg/ml of PHA and maintained with 50 U/ml IL-2 and 10 % FBS in RPMI-1640 medium for three days prior to HIV-1 infection. HIV-1_IIIB_, HIV-1_MN_, and HIV-1_RF_ were propagated in C8166 cells; HIV-1 primary isolates-HIV-1_KM018_, HIV-1_WAN_, and HIV-1_TC-2_ were propagated in PBMCs.

### Cytotoxicity

The cell viability of SJP-L-5 was determined by the MTT method as previously described [71]. Briefly, C8166 cells were diluted in fresh medium to a final concentration of 4 × 10^5^/ml and added to a 96-well plate at 100 μl/well, and then a series of concentrations of SJP-L-5 were added to each well (100 μl). After 3 days of incubation at 37 °C, 5 % CO_2_, the cell viability was determined using MTT; for PBMCs, 5 × 10^5^ cells were added to each well and the plates were incubated for 7 days. The percentage of cell viability was compared to that of the DMSO solvent control. Afterward, the CC_50_ was calculated by sigmoidal fit using Origin Pro 7.5 software based on the cell viability in the presence and absence of the compound tested. AZT was used as a reference compound. The data presented are representative of three independent experiments.

### Antiviral activity

Antiviral activity was assessed by ELISA as previously described [72]. C8166 cells were infected by HIV-1_IIIB_, HIV-1_MN_, or HIV-1_RF_ at a multiplicity of infection (MOI) of 0.03 in the presence of different serial concentration of SJP-L-5 as described previously [73]. After a 2 h infection at 37 °C in a 5 % CO_2_ atmosphere, HIV-1-infected C8166 cells were washed three times with phosphate-buffered saline (PBS) to remove free viruses. Next, the infected cells were resuspended in RPMI-1640 and seeded into a 96-well plate (4 × 10^4^ in 100 μl of RPMI-1640 per well) with 100 μl of gradient concentrations of SJP-L-5. AZT was used as a reference compound. On day 3 post infection, p24 levels were measured by an in-house ELISA, and the EC_50_ was calculated by sigmoidal fit using Origin Pro 7.5 software. For PBMCs, cells were infected with HIV-1_KM018_, HIV-1_WAN_, and HIV-1_TC-2_ at low MOIs (0.06–0.1) for 2 h. Infected PBMCs were then washed three times with PBS, after which 100 μl of 5 × 10^5^ infected cells were seeded into each well of a 96-well plate in the presence of a gradient of different concentrations of SJP-L-5. On day 7 post infection, p24 antigen levels were measured by ELISA, and the EC_50_ was calculated as described above. The data presented are representative of three independent experiments.

### Generation of VSV-G pseudotyped viruses

pMD.G, pCMV∆R8.2, pTRIP-GFP, pNL4-3-Luc, pCMV-VSV-G, pHIT60, and pMA-Luc plasmids were provided by Professor Guangxia Gao (Institute of Biophysics, Chinese Academy of Sciences). pMD.G was used to generate the stomatitis virus G (VSV-G) envelope [74]. pCMV∆R8.2 is an encapsidation plasmid that contains the HIV-1 *gag-pol* gene, as well as some accessory protein genes [75]. pTRIP-GFP was derived from pHR’-CMV LacZ, in which *lacZ* reporter gene was replaced by enhanced GFP (eGFP) [76]. pNL4-3-Luc contains a luciferase reporter, with the HIV-1 envelope gene sequence mutated [77]. MLV-Luc-producing plasmids pCMV-VSVG, pHIT60, and pMA-Luc have been previously described [17, 78].

293 T cells were used to generate VSV-G pseudotyped HIV-1 or MLV. All retroviral vectors were pseudotyped with VSV-G to maintain one round of infection and produced by transient transfection into 293 T cells using Lipofectamine 2000 reagent (Invitrogen, Carlsbad, CA, USA). For HIV-1-GFP reporter virus (HIV-1-GFP) production, the proviral plasmid pTRIP-GFP, the encapsidation plasmid pCMV∆R8.2, and the VSV-G envelope expression plasmid were used to cotransfect into 293 T cells at approximately 90 % confluency, following the manufacturer’s instructions. For HIV-1 luciferase reporter virus (HIV-1-Luc) production, the plasmid pNL4-3-Luc with the HIV-1 envelope gene sequences deleted, and the VSV-G envelope expression plasmid were used to co-transfect into 293 T cells as described above. To produce VSV-G pseudotyped MLV-Luc, pCMV-VSV-G, pHIT60, and pMA-Luc were transfected into 293 T cells. The supernatants containing the virus were harvested at 36, 48, 60, and 72 h post transfection and treated with DNase I (1 mg/ml in the presence of 10 mM MgCl_2_) for 60 min at 37 °C, followed by passage through a 0.45-μM filter to remove cell debris. Virus stocks were stored at −80 °C. 10-fold dilutions of the virus were used to infect 293 T cells.

### Single cycle infectivity assays and anti-HIV-1 activity of SJP-L-5

293 T cells were seeded in dishes the day before infection. After 24 h, the cells were incubated with recombinant viruses and 8 μg/ml polybrene, in the presence or absence of SJP-L-5 at 37 °C in a 5 % CO_2_ atmosphere. The virus supernatant was removed and replaced with fresh medium 3 h post-viral infection, and SJP-L-5 was maintained at all times; SJP-L-5 was dissolved in DMSO. The final concentration of DMSO in the cell culture medium was kept at less than 0.3 % to minimize the cytotoxicity of the solvent.

To test luciferase activities, the cells were washed once with cold PBS 48 h after infection and lysed in reporter lysis buffer (Promega, Madison, WI, USA) for 10 min on ice. The cell lysates were centrifuged at 12,000 *g* for 5 min at 4 °C. Luciferase activity was determined by adding 100 μl of substrate to 20 μl of cell lysate using a luminometer (Promega, Model TD-20/20). The data presented are representative of three independent experiments.

### Hirt DNA extraction

To quantify viral DNA products by real-time PCR, low-molecular-weight DNA was extracted using the Hirt extraction method [79, 80]. Briefly, 293 T cells were washed with ice-cold PBS and lysed using lysis buffer (0.6 % SDS, 10 mM EDTA, 100 mM Tris–HCl, pH 7.5) at room temperature for 10 min. Then 5 M NaCl was added and incubate at 4 °C overnight. The mixture was centrifuged at 4 °C for 30–60 min. The DNA was extracted with phenol/chloroform/isoamyl alcohol (25:24:1) and precipitated by adding 2.5 volumes of ethanol. The DNA was resuspended in Tris-EDTA (TE) buffer and stored at −20 °C.

### Real-time PCR

Real-time PCR assays were performed using the SYBR Green Universal PCR Master Mix kit (TOYOBO, Tokyo, Japan), and the products were quantified using the delta-delta Ct method, which were performed using the Applied Biosystems ABI Prism 3000 sequence detection system (Thermo Fisher Scientific, Waltham, MA, USA).

All reactions were conducted in a total volume of 20 μl. To normalize the quantity of total cellular DNA present in each sample, human mitochondrial DNA was amplified and used as an internal control. Human mitochondria have a circular episomal DNA about 16.5 kbp in length. A few hundred copies of mitochondrial DNA (mtDNA) are present in every cell. The primers used in this paper were as follows (Table 2) according to a previous report [81]: M-F and M-R were used for the amplification of -sssDNA. P-F and P-R were used for flDNA; CIR-F and CIR-R for Circular DNA; and Mito-F and Mito-R for human mitochondrial DNA. AZT was used as a positive control. The data presented are representative of three independent experiments.

**Table 2:** Primers used to detect HIV-1 DNA products

Primer	Sequence	Position
M-F	TTAGACCAGATCTGAGCCTGGGAG	R-U5
M-R	GGGTCTGAGGGATCTCTAGTTACCAC	R-U5
P-F	TGTGTGCCCGTCTGTTGTG	U5-gag
P-R	GAGTCCTGCGTCGAGA	U5-gag
CIR-F	CCCTCAGACCCTTTTAGTCAGTG	U5-U3 in 2-LTR circle
CIR-R	TGGTGTGTAGTTCTGCCAATCA	U5-U3 in 2-LTR circle
Mito-F	CCACTTTCCACACAGACATC	
Mito-R	TCTGGTTAGGCTGGTGTTAG	

### Transient transfections and subcellular localization studies

1 × 10^5^ 293 T cells were plated on coverslips in a 24-well plate. Transient transfection was performed using Lipofectamine 2000 reagent (Invitrogen) when cells were approximately 90 % confluent. 0.2 μg of the abovementioned plasmids above was transfected according to the manufacturer’s instructions. After 48 h, the cells were fixed with 4 % paraformaldehyde (w/v), and stained with 4′, 6-diamidina-2-phenylindole (DAPI) according to the manufacturer’s protocol. The subcellular localization of the fusion proteins was analyzed by detecting GFP using a confocal microscope (PV1000-IX81, Olympus, Tokyo, Japan).

### Fate-of-capsid assay

Approximately 6 × 10^6^ 293 T cells were incubated with 4 ml of undiluted recombinant viruses at 37 °C. At each time point after infection, the cells were harvested for the sedimentation assay. Briefly, the cells were washed with ice-cold PBS and detached by pipetting up and down. Then, the cells were resuspended in 1.5 ml of hypotonic lysis buffer (10 mM Tris–HCl, pH 8.0, 10 mM KCl, 1 mM EDTA, and one Complete Protease Inhibitor tablet (Roche, Basel, Switzerland)), and incubated on ice for 15 min. The cells were lysed using 20 passes in a 2-ml Dounce homogenizer with pestle B. The lysate was centrifuged at 800 g (KUBOTA, Tokyo, Japan) for 5 min to remove cell debris. After centrifugation, 100 μl of the cleared lysate was collected and mixed with 2× sodium dodecyl sulfate (SDS) sample buffer, which was referred to as the p24 input fraction. 1 ml of the cleared lysate was layered onto a 50 % sucrose (w/v) cushion in PBS and centrifuged at 125,000 *g* for 2 h at 4 °C in a P40ST rotor (Hitachi, Tokyo, Japan). After centrifugation, 100 μl of the uppermost part of the supernatant was collected and mixed with 2× SDS sample buffer; this part was referred to as the p24 soluble fraction. The pellet was resuspended in 50 μl of 1× SDS sample buffer and referred to as the p24 particulate fraction; β-Actin was used as a pellet fraction control. All samples were then subjected to SDS-PAGE and Western blotting. The HIV-1 p24 CA protein was detected using a mouse monoclonal antibody purified by the laboratory of Professor Yongtang Zheng. The data presented are representative of three independent experiments.

### Transmission electron microscopy

293 T cells were seeded in dishes the day before infection. After 24 h, the cells were incubated with VSV-G pseudotyped HIV-1 and polybrene at a final concentration of 8 μg/ml in the presence or absence of SJP-L-5 at 37 °C. The virus supernatant was removed and replaced with fresh medium 4 h post-viral infection, and SJP-L-5 was maintained at all times. At 4 h and 8 h post infection, the cells were harvested for the TEM assay. Cells were fixed for 12 h at 4 °C in 2.5 % glutaraldehyde in 0.1 M phosphate buffer (pH 7.3), washed, fixed again in aqueous 1 % osmium tetroxide, and embedded in EPON. TEM was performed with a Tecnai G2 Spirit-120 kV transmission electron microscope (FEI, Hillsboro, OR, USA), at 120 kV, on ultrathin sections (80-nm thick) stained with uranyl acetate and lead citrate. 20 cells per field were selected randomly in the control and SJP-L-5 groups. Quantification of total HIV-1 was performed on 20 fields in the control and SJP-L-5 groups.

### Statistical analysis

Results were presented as mean ± S.D. of three independent experiments. The differences between control and target data sets was tested by Student’s *t* test, and P-values ≤ 0.05 were considered to be of statistical significance.

## Additional files

Additional file 1:
**Primers used in plasmid constructions.** **Table S1.** Primers used in plasmid constructions. (DOCX 18 kb) Additional file 2:
**Subcellular localization of** **HIV-1** **IN, MA, and Vpr in transfected cells.** **Figure S1.** Subcellular distributions of HIV-1 IN, MA and Vpr in transfected cells. (A). Subcellular distributions of HIV-1 IN, MA, and Vpr in control cells. 293 T cells were transiently transfected with 0.2 μg of the plasmids pC1-GFP, pC1-GFP_2_-NLS, pGFP-IN, pGFP-MA, or pGFP-Vpr using Lipofectamine 2000 reagent, the primers used in plasmid constructions were shown in Additional file 1: Table S1. At 48 h post transfection, the cells were fixed with 4 % paraformaldehyde (w/v) and then stained with DAPI. (B-D). Subcellular distributions of HIV-1 IN, MA, and Vpr in SJP-L-5 treated cells. 293 T cells were transiently transfected with 0.2 μg of the plasmids pGFP-IN (B), pGFP-MA (C), or pGFP-Vpr (D) using Lipofectamine 2000 reagent in the presence of 5 μg/ml or 20 μg/ml of SJP-L-5. At 48 h post transfection, the cells were fixed with 4 % paraformaldehyde (w/v) and then stained with DAPI. GFP (Green) and DAPI (Blue) fluorescence were observed using a confocal microscope. (TIFF 1744 kb) Additional file 3:
**The effect of** **SJP-L-5** **on the** **HIV-1** **DNA flap.** **Figure S2.** The effect of SJP-L-5 on the HIV-1 DNA flap. 293 T cells were infected with VSV-G pseudotyped NL4-3-Luc or CMVΔR8.2-Luc in the presence of SJP-L-5 (5 μg/ml, Gray) or DMSO (Black) as a negative control. The antiretroviral activity was evaluated by testing the activity of the luciferase reporter gene at 48 h post infection. Error bars indicate standard deviations of triplicate values. Statistical significance was analyzed by the Student’s *t* test. ^***^ *P* < 0.001 versus the negative control. (TIFF 370 kb)

## Abbreviations

HIV-1: Human immunodeficiency virus, type1
MLV: Murine leukemia virus
PIC: Pre-integration complex
NPC: Nuclear pore complexes
CA, MA, IN, Vpr: The capsid, matrix, integrase, and viral protein regulatory of HIV-1, respectively
PBMC: Peripheral blood mononuclear cell
NLS: Nuclear localization signal
sssDNA: single strand strong-stop DNA
LTR: Long terminal repeat
TEM: Transmission electron microscopy
MOI: Multiplicity of infection
CC_50_: 50 % cytotoxicity concentration
EC_50_: 50 % effective concentration
