# Supplementary material for: SJP-L-5, a novel small-molecule compound, inhibits HIV-1 infection by blocking viral DNA nuclear entry
Source: BMC Microbiol. 2015 Dec 2;15:274. doi: 10.1186/s12866-015-0605-3 (PMC4667461; doi:10.1186/s12866-015-0605-3)
Supplement: Additional file 1: — Primers used in plasmid constructions. Table S1. Primers used in plasmid constructions. (DOCX 18 kb) [file 12866_2015_605_MOESM1_ESM.docx]

**Additional file 1 – Primers used in plasmids construction.**

Table S Primers used in plasmids construction

| ***Primer*** | ***Sequence*** |
| --- | --- |
| IN-F | CCGCTCGAGACCATGTTTTTAGATGGAATA |
| IN-R | TCCCCGCGGATCCTCATCCTGTCTACT |
| MA-F | CCGCTCGAGACCATGGGTGCGAGAGCGTCA |
| MA-R | TCCCCGCGGGTAATTTTGGCTGACCTG |
| Vpr-F | CCGCTCGAGACCATGGAACAAGCCCCAGAA |
| Vpr-R | TCCCCGCGGGGATCTACTGGCTCCATT |
