# Supplementary figures and images for: SJP-L-5, a novel small-molecule compound, inhibits HIV-1 infection by blocking viral DNA nuclear entry
Source: BMC Microbiol. 2015 Dec 2;15:274. doi: 10.1186/s12866-015-0605-3 (PMC4667461; doi:10.1186/s12866-015-0605-3)

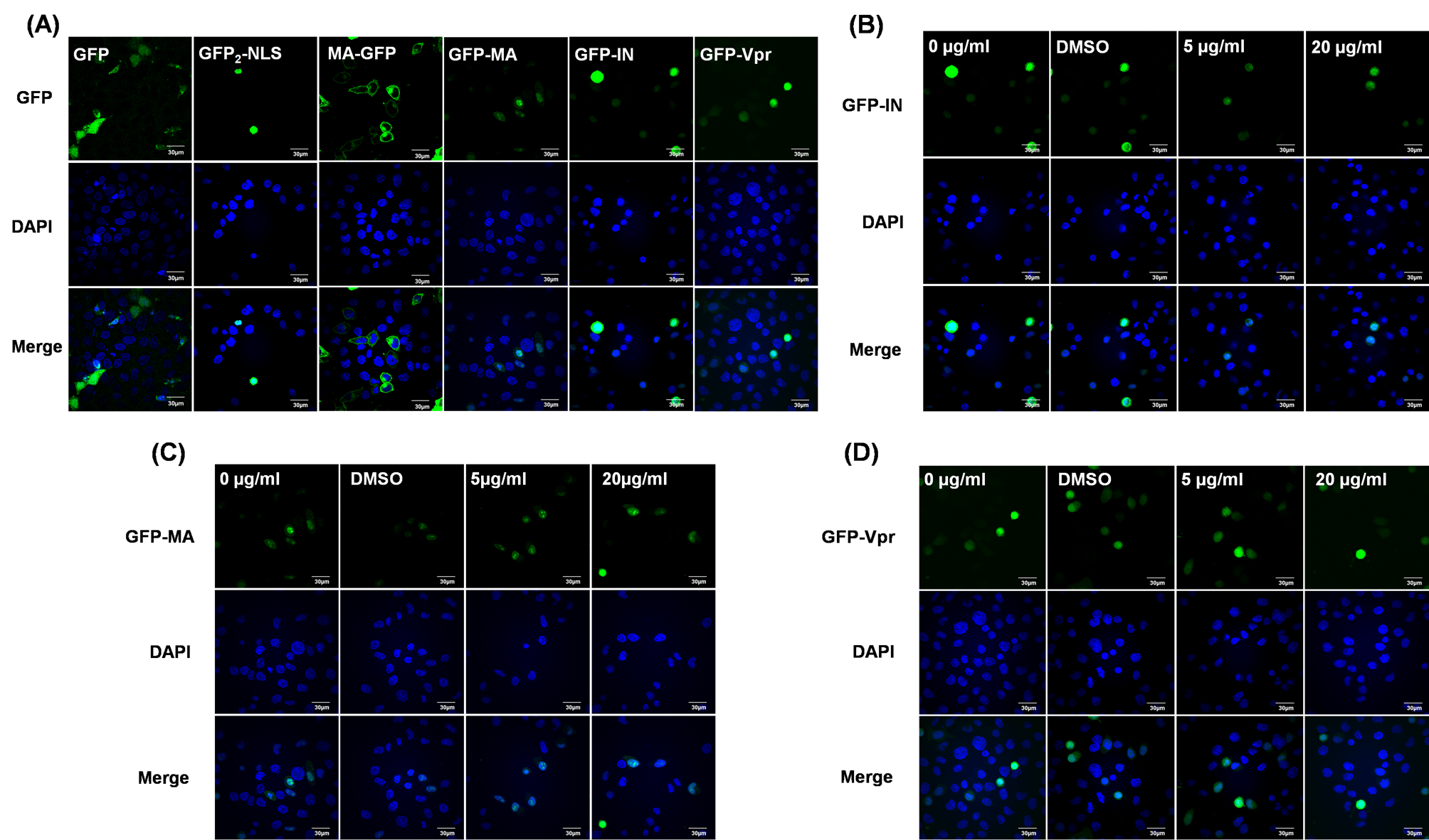

Supplement: Additional file 2: — Subcellular localization of HIV-1 IN, MA, and Vpr in transfected cells. Figure S1. Subcellular distributions of HIV-1 IN, MA and Vpr in transfected cells. (A). Subcellular distributions of HIV-1 IN, MA, and Vpr in control cells. 293 T cells were transiently transfected with 0.2 μg of the plasmids pC1-GFP, pC1-GFP2-NLS, pGFP-IN, pGFP-MA, or pGFP-Vpr using Lipofectamine 2000 reagent, the primers used in plasmid constructions were shown in Additional file 1: Table S1. At 48 h post transfection, the cells were fixed with 4 % paraformaldehyde (w/v) and then stained with DAPI. (B-D). Subcellular distributions of HIV-1 IN, MA, and Vpr in SJP-L-5 treated cells. 293 T cells were transiently transfected with 0.2 μg of the plasmids pGFP-IN (B), pGFP-MA (C), or pGFP-Vpr (D) using Lipofectamine 2000 reagent in the presence of 5 μg/ml or 20 μg/ml of SJP-L-5. At 48 h post transfection, the cells were fixed with 4 % paraformaldehyde (w/v) and then stained with DAPI. GFP (Green) and DAPI (Blue) fluorescence were observed using a confocal microscope. (TIFF 1744 kb) [file 12866_2015_605_MOESM2_ESM.tiff]

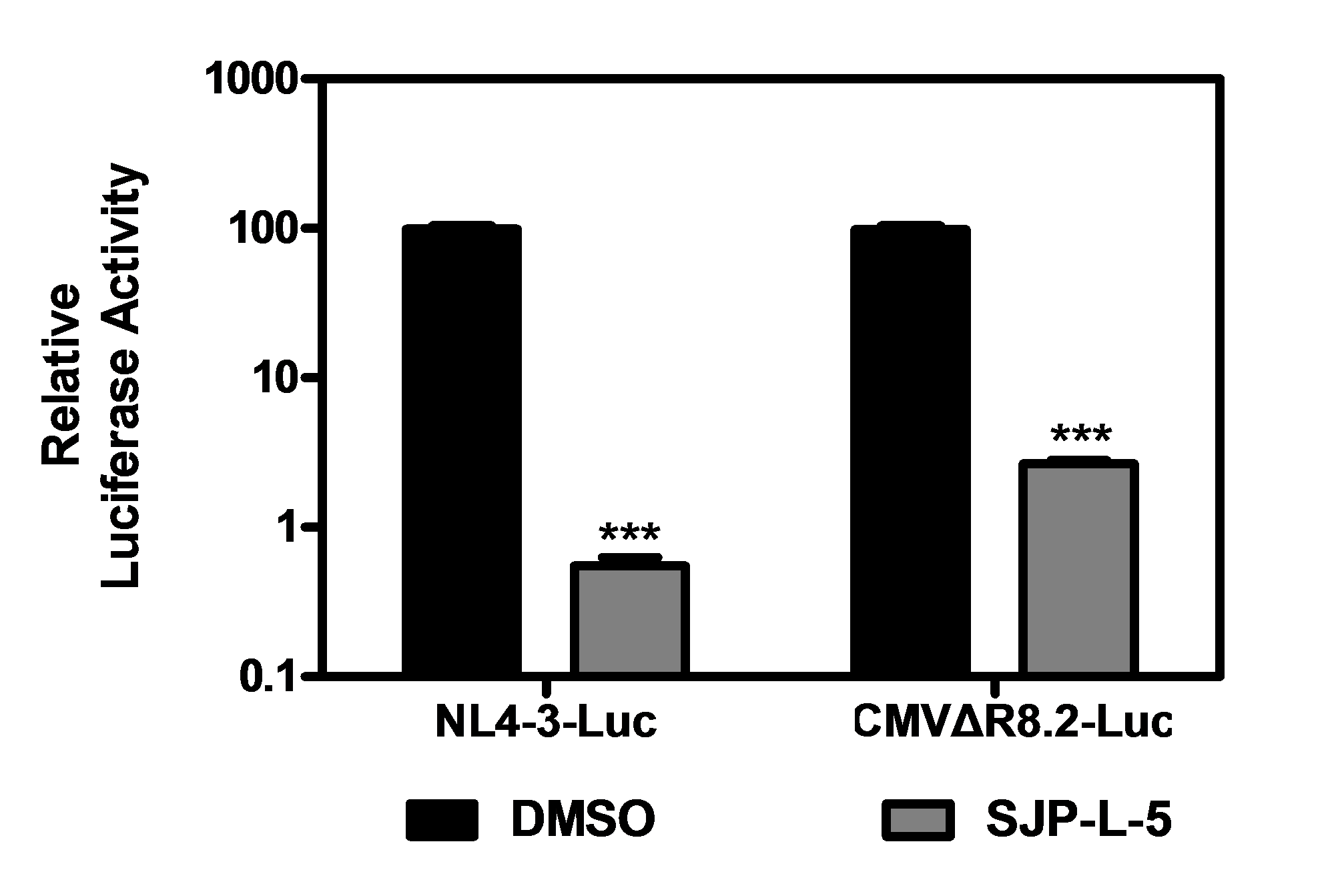

Supplement: Additional file 3: — The effect of SJP-L-5 on the HIV-1 DNA flap. Figure S2. The effect of SJP-L-5 on the HIV-1 DNA flap. 293 T cells were infected with VSV-G pseudotyped NL4-3-Luc or CMVΔR8.2-Luc in the presence of SJP-L-5 (5 μg/ml, Gray) or DMSO (Black) as a negative control. The antiretroviral activity was evaluated by testing the activity of the luciferase reporter gene at 48 h post infection. Error bars indicate standard deviations of triplicate values. Statistical significance was analyzed by the Student’s t test. *** P < 0.001 versus the negative control. (TIFF 370 kb) [file 12866_2015_605_MOESM3_ESM.tiff]
